# Expression of Myosin Heavy Chain and Some Energy Metabolism-Related Genes in the Longissimus Dorsi Muscle of Krškopolje Pigs: Effect of the Production System

**DOI:** 10.3389/fvets.2020.533936

**Published:** 2020-09-18

**Authors:** Gregor Fazarinc, Milka Vrecl, Klavdija Poklukar, Martin Škrlep, Nina Batorek-Lukač, Jana Brankovič, Urška Tomažin, Marjeta Čandek-Potokar

**Affiliations:** ^1^Veterinary Faculty, Institute of Preclinical Sciences, University of Ljubljana, Ljubljana, Slovenia; ^2^Animal Science Department, Agricultural Institute of Slovenia, Ljubljana, Slovenia

**Keywords:** Krškopolje pigs, production system, skeletal myofibers, contractile phenotype, metabolic phenotype, histochemistry, qPCR

## Abstract

The Slovenian Krškopolje pig is the only preserved local autochthonous breed, appreciated mainly for its good meat quality and considered more appropriate for processing into dry-cured products. However, the biological characteristics of the skeletal myofibers of the Krškopolje breed, specifically the heavy myosin chain-based contractile and metabolic phenotypes that could affect meat quality, have not been established under different husbandry systems. The breed is generally maintained in either conventional indoor or organic systems. In the present study, the morphological, contractile, and metabolic properties of myofibers of the longissimus dorsi muscle were compared between animals reared in either an organic or a conventional indoor system. The myofibers were studied using immunohistochemical and succinate dehydrogenase (SDH) activity-based classification, histomorphometric assessment, and qPCR. Results revealed that the organic production system influenced the composition of the longissimus dorsi myofiber type, characterized by a smaller myofiber cross-sectional area, a shift toward oxidative (SDH-positive) myofiber types, increased relative expression of myosin heavy chain (MyHC) isoforms I, IIa, and IIx, and downregulation of MyHC IIb. On the contrary, no apparent effect was observed on the metabolic phenotype of the myofiber as assessed through relative mRNA expression of energy metabolism-related genes [peroxisome proliferator-activated receptor gamma, coactivator 1 alpha *(PGC-1*α*)*, peroxisome proliferator-activated receptor gamma *(PPAR*γ*)*, lipoprotein-lipase *(LPL)*, carnitine palmitoyltransferase 1B *(CPT1B)*, glycogen synthase 1 *(GYS1)*, hexokinase 2 *(HK2)*, and fatty acid synthase *(FASN)*]. Differences in MyHC expression were largely corroborated by the histochemical classification, indicating that the contractile protein content is directly regulated by the MyHC genes. A correlation between the muscle contractile and metabolic phenotypes was not established, except for that between the *HK2* and *MyHC I* genes. In conclusion, the present study showed an evident effect of rearing on the longissimus dorsi myofiber contractile phenotype but not the metabolic phenotype. Moreover, obtained data suggest that rearing the Krškopolje pig breed in a conventional system would result in an increased fiber size and a greater proportion of type IIb myofibers, which are known to be negatively correlated with some meat quality traits.

## Introduction

Myofibers constitute 75–90% of skeletal muscle and are largely responsible for the determination of meat quality traits, in particular, through morphological and physiological characteristics (1–3). In pig skeletal muscles, myofibers are classified into four different types, including I, IIa, IIx, and IIb. They are characterized by the myosin heavy chain (MyHC) isoform expression, with different shortening velocities increasing in the order MyHC I < IIa < IIx < IIb, and MyHC expression can shift from MyHC I to IIa, IIa to IIx, and IIx to IIb reversibly (4, 5). Consequently, hybrid myofibers that contain more than one MyHC isoform, are also present in muscles. The metabolic profile of myofibers generally meets the energetic demands of the MyHC isoform. High oxidative capacity is a characteristic feature of type I, which have a higher mitochondria and myoglobin content, with lipids as the main energetic source. The IIb myofibers have higher glycogen content for quick, short-lasting contractions, while type IIa and IIx myofibers represent a metabolically intermediate type (6). Type IIa myofibers have higher oxidative capacity than type IIx myofibers (3, 6). In pigs, reprogramming of the myofiber phenotype alters the contractile and metabolic properties of the muscle and can consequently influence meat quality. Such an impact on myofiber phenotype can appear due to various factors, including selective breeding, rearing conditions, dietary regime, or physical activity (7–10).

For decades, pig breeding programs have focused on improving the growth rate, feed efficiency, and carcass lean meat content (11). This has resulted in myofiber hypertrophy, an increased proportion of IIb myofibers, and a switch to higher glycolytic muscle metabolism in modern pig breeds, thereby causing some undesirable impacts on meat quality (12). On the contrary, autochthonous (local) pig breeds have been neglected since many years and have not been selected for growth rate and muscle tissue deposition. Moreover, these breeds are usually reared in production systems characterized by more welfare-friendly conditions. They exhibit slower growth rates and higher body fatness (13), and their products are of better quality compared to modern, highly selected pig breeds (14).

Manipulating the muscle myofiber type composition through physical activity, nutrition, and environmental rearing conditions have become of utmost important because of growing interest in organic rearing and concerns about animal welfare. The Krškopolje pig breed is the only autochthonous pig breed in Slovenia that is reared in a variety of husbandry systems, but mainly in a more free-range way. This breed has originated from southeastern Slovenia and has been relatively poorly studied. Various reports have indicated limited muscular development and increased fat deposition in this breed (15). It is considered more suitable for products like dry-cured ham (16) than modern leaner pig breeds, including Landrace, Large White, and Pietrain. Results of our recent study obtained from the Krškopolje breed have demonstrated an important effect of organic vs. conventional rearing on fat and meat quality traits, such as pH, lipid oxidation status, and fatty acid composition (longissimus dorsi muscle of organically reared pigs contained a higher proportion of monounsaturated fatty acids) (17). However, the interaction between some genes related to muscle contractile phenotype/energy metabolism, including MyHCs and peroxisome proliferator-activated receptor gamma coactivator 1 alpha (PGC-1α), and their consequences under different husbandry systems has not been established. Therefore, it has been hypothesized that the production system could impact the contractile and metabolic properties of the longissimus muscle of Krškopolje pigs, in particular, on the myofiber types composition and their hypertrophic potential. The present study aimed to test this hypothesis by analyzing the effects of organic outdoor vs. conventional indoor production systems on the morphological, contractile, and metabolic properties of longissimus muscle myofibers of Krškopolje pigs. Furthermore, correlation between the relative expression of MyHCs and selected energy metabolism-related genes that define muscle tissue contractile and metabolic properties was also investigated.

## Materials and Methods

### Animals and Rearing Conditions

Animals and rearing conditions were selected as previously described (17). Briefly, 24 barrows originating from 12 L were assigned within litter to either conventional (C; *n* = 12) or organic (O; *n* = 12) group. Both groups were reared in the same farm; however, the organic group was reared in compliance with the Commission regulation (889/2008) for organic production, with a sheltered area of 16 m^2^ and an outdoor paddock area of 100 m^2^. Pigs of the group C were housed indoors (7.5 m^2^/pen). Pigs were fed a barley-based feed mixture, equivalent for the C and O groups of pigs in terms of energy, nutrient content, and fatty acid composition (17). However, the diet of O pigs was composed of organically grown ingredients. In line with organic farming, pigs of the O group were also offered roughage (alfalfa hay). In the beginning of the trial, the pigs were weighted 68 ± 8 kg at the age of 157 ± 6 days. After a period of 73 days, pigs were sent to slaughter (in a commercial abattoir according to the standard procedure, with CO_2_ stunning followed by de-hairing, evisceration, and veterinary inspection). There were no significant differences between pigs from the C and O groups in carcass mass (97.6 vs. 98.8 kg, respectively) and longissimus dorsi area (37.0 vs. 35.6 cm^2^, respectively) (17).

### Immunoenzyme Histochemistry of Muscle Samples

Muscle samples were collected from the central part of the longissimus dorsi muscle (LD) at the level of the last thoracic vertebrae within 2 h after slaughter, deep-frozen in liquid nitrogen, and stored at −80°C till further analysis. Immunoenzyme histochemistry was performed on a subset of animals (6 animals per group) as previously described (18). Briefly, three different monoclonal antibodies (MAb) specific for adult MyHC isoforms were used: NLC-MHCs, specific for slow-twitch MyHC I; SC 71, specific for MyHC IIa; and BF-F3, specific for rat MyHC IIb (19). The reactivities of the listed MAbs for pig myofiber classification have been previously confirmed (6, 20). Transverse serial cryosections (10 μm) of muscle tissues cut with a cryostat Leica CM 1800 (Heidelberg, Germany) at −17°C and mounted on Thermo Scientific™ Superfrost® Plus adhesion slides (Gerhard Menzel B.V. & Co. KG, Braunschweig, Germany) were incubated with the primary antibody in a humidified chamber at 4°C overnight. The immunohistochemical reaction was visualized using a peroxidase-conjugated secondary antibody kit using the Dako REAL™ DAB chromogen (Copenhagen, Denmark). The oxidative capacity of the myofibers was estimated on the basis of the activity of mitochondrial succinate dehydrogenase (SDH) (21). The sections were dehydrated and mounted using Synthetic Mountant (Shandon, CA, USA). Classification of myofiber types of pig longissimus dorsi muscles into I, IIa, IIx, and IIb was based on a previous study (6) and is summarized in Table 1. Type I myofibers displayed the strongest SDH activity, denoted as ++; type IIa displayed strong/moderate SDH activity, denoted as ++/+, respectively; type IIx displayed moderate/negative SDH activity, denoted as +/–, respectively; and IIb displayed predominantly negative SDH activity, denoted as - Table 1.

**Table 1:** Specificity/intensity of immunohistochemical staining and the succinate dehydrogenase (SDH) activity supporting the classification of myofiber types into I, IIa, IIx, and IIb.

		**Myofiber type**			
		**I**	**IIa**	**IIx**	**IIb**
Antibody	NLC-MHCs	++	–	–	–
	SC 71	–	++	+	–
	BF-F3	–	–	+/–	++
SDH		++	++/+	+/–	–

*+ and ++ denote a moderate and strong positive reactions, respectively. – denotes a negative reaction*.

The histomorphometric analysis of the transverse serial sections was performed using a Nikon Eclipse Ni-UM microscope equipped with a DS-Fi1 camera and the Imaging Software NIS-Elements BR 4.60 (Nikon instruments Europe B.V., Badhoevedorp, The Netherlands) as previously described (18). Approximately 500 myofibers in randomly selected complete muscle fascicles were analyzed per muscle sample to determine the average proportion of individual myofiber types according to the immunohistochemical staining and SDH activity as well as the cross-sectional area (CSA) of myofiber types. To assess the oxidative capacity of the LD muscle, the glycolytic-to-oxidative myofiber and relative area ratios were calculated. Glycolytic-to-oxidative myofiber ratio was calculated by dividing the proportion of type IIb myofibers by the proportion of type I, IIa, and IIx myofibers. Glycolytic-to-oxidative relative area was calculated by dividing the relative area ratio of type IIb myofibers by the relative area ratio of type I, IIa, and IIx myofibers.

### RNA Isolation, cDNA Synthesis, and Quantitative Polymerase Chain Reaction (qPCR)

RNA isolation from frozen LD samples, cDNA synthesis, and qPCR were performed as previously described (22). Briefly, total RNA was extracted from ~25 mg frozen muscle tissue using an RNeasy Fibrous Tissue Mini Kit (Qiagen, Stockach, Germany) and subjected to an on-column DNase digestion step using the RNase-Free DNase Set (Qiagen, Stockach, Germany). The 260/280 and 260/230 absorbance ratios were then determined using UV–VIS Lambda 25 spectrophotometer (Perkin Elmer, Waltham, MA, USA) to quantify the extracted RNA and check for potential contamination. Thereafter, three out of the 24 RNA samples, one extracted from the C, and two from the O group muscle samples, were excluded due to insufficient low RNA quantity/quality. Subsequently, 1 μg of each RNA sample with 260/280 and 260/230 ratios close to 2.0 was used for cDNA synthesis using the RT^2^ First Strand Kit (Qiagen, Stockach, Germany). Primers and fluorescent 6-FAM dye-labeled minor groove binder probes/predesigned assays were obtained from Applied Biosystems (Thermo Scientific GmbH, Vienna, Austria). Primers/probes to detect the MyHC isoforms I, IIa, IIx, and IIb, reflecting muscle contractile phenotype, were used as reported previously (22). Information on the pre-developed assays used for assessing the muscle metabolic phenotypes and their prospective functions, using two endogenous controls including the eukaryotic ribosomal (r) 18S RNA (18S rRNA) that displays a high level of conservation amongst eukaryotes and actin beta (*ACTB*), are presented in Table 2. The 18S rRNA and *ACTB* has been previously shown as suitable controls for qPCR data normalization of LD muscle samples in pig (23, 24). The mean cycle threshold (Ct) values for 18S rRNA in the C and O group of pigs were 13.69 ± 0.63 and 14.39 ± 0.77, respectively. The mean Ct values for *ACTB* in the C and O group of pigs were 28.30 ± 0.46 and 28.09 ± 0.51, respectively.

**Table 2:** List of predesigned TaqMan gene expression assays used for qPCR.

**Full gene name**	**Gene**	**Amplicon length**	**Assay ID**	**Function/use**
Peroxisome proliferator-activated receptor gamma, coactivator 1 alpha	*PGC-1α*	96	Ss03393114_u1	Regulation of genes involved in energy metabolism
Peroxisome proliferator-activated receptor gamma	*PPAR*γ	72	Ss03394829_m1	Fatty acid storage and glucose metabolism
Lipoprotein-lipase	*LPL*	66	Ss03394612_m1	Triglyceride hydrolysis
Carnitine palmitoyltransferase 1B	*CPT1B*	60	Ss03378792_u1	Transport of long-chain fatty acyl-CoAs into mitochondria
Glycogen synthase 1	*GYS1*	101	Ss03376867_u1	Glycogen synthesis
Hexokinase 2	*HK2*	78	Ss03390132_m1	Glycolysis
Fatty acid synthase	*FASN*	95	Ss03386194_u1	Fatty acid synthesis
Actin, beta	*ACTB*	77	Ss03376081_u1	Endogenous control
Eukaryotic ribosomal (r) 18S rRNA	*18S rRNA*	69	Hs03003631_g1	Endogenous control

qPCR was carried out in 96-well-plates with a final reaction volume of 10 μL consisting of 4.5 μL of the 10-fold diluted cDNA sample and 5.5 μL of the TaqMan universal PCR Master Mix in the QuantStudio™ 5 Real-Time PCR System (ThermoFisher Scientific, Applied Biosystems, Foster City, CA, USA). The following conditions were used for PCR amplification: one cycle of 50°C for 2 min and one cycle of 95°C for 10 min followed by 45 cycles of 15 s at 95°C and 1 min at 60°C. Each reaction was run in triplicates. Briefly, the results were calculated from a threshold cycle (Ct) at which the PCR product crossed the detection threshold. The threshold line that showed the threshold cycle number (Ct) was fixed at 0.10. A Ct value >40 was defined as the cutoff (for the detection of gene expression). Relative quantification of target transcripts, normalized against the geometric mean of 18S rRNA and *ACTB*, was performed according to the comparative Ct method (ΔCt = Ct_geometric mean of controls_ – Ct_target transcript_), and the relative changes in the expression of the studied target transcripts (fold changes in the expression) between indoor conventional (C) or organic (O) group of pigs was determined using the 2^ΔΔCt^ method. The PCR efficiency of studied genes was >90% and was derived from standard curves composed of four 10-fold dilutions of the cDNAs. Applied Biosystems™ Analysis Software, Relative Quantification Analysis Module, version 3.9, was used for data analysis.

### Statistical Analysis

To compare the expression of contractile and energy metabolism genes under organic (O) vs. conventional husbandry (C), analysis of variance (procedure GLM of SAS/STAT® software; SAS Institute Inc., Cary, NC, USA) was used with the model, including the fixed effect of treatment group (O vs. C). In the case of a significant effect (*p* < 0.05), means were compared using the Tukey test. The magnitude of differences between treatment groups, such as effect size, was also assessed with Cohen's d (25). To determine and visualize the relationship between MyHC mRNA expression and myofiber typing, and between contractile and energy metabolism gene expression, principal component analysis (PCA) was performed using R software (26). FactoMineR (27) was used for PCA calculations, while the Factoextra package (28) was used for visualizing the graph of variables. Pearson correlation coefficient (r) was computed using the Hmisc package (29), and the correlation matrix was displayed using the Corrplot package (30).

## Results

### Myofiber Types Composition and Morphological Characteristics

**Figure 1:**
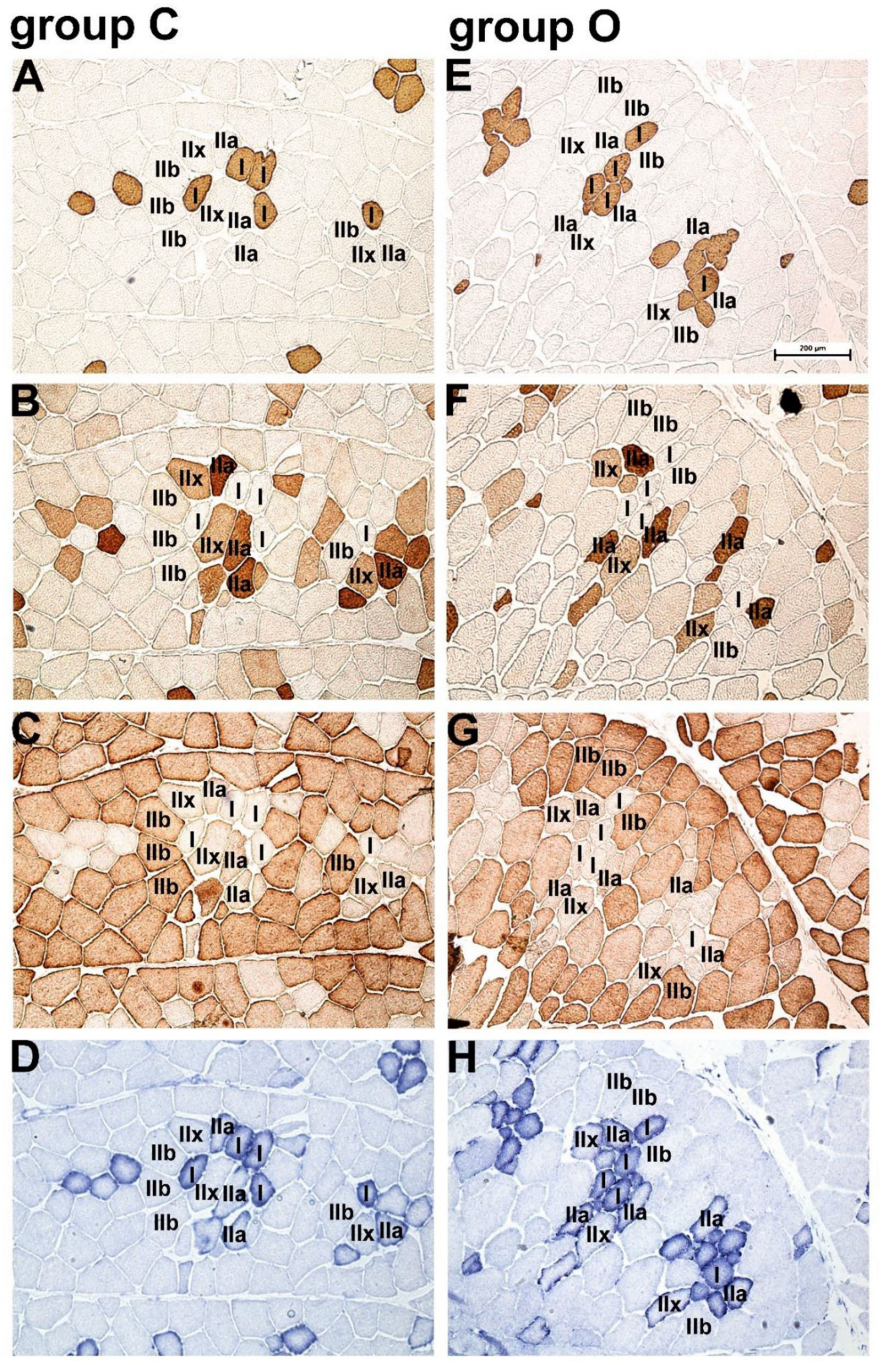
Immunohistochemical and succinate dehydrogenase (SDH) activity-based classification of myofiber types in Krškopolje pig longissimus dorsi (LD) muscle. **(A)** Serial transverse sections of the LD muscle from pigs raised in the conventional indoor husbandry system (group C; **A–D**) or in organic conditions (group O; panels **E–H**) stained with the monoclonal antibodies **(A,E)** NLC-MHCs, **(B,F)** SC 71, and **(C,G)** BF-F3, specific for myofiber types I, II, and IIb, respectively; **(D,H)** succinate dehydrogenase (SDH) activity demonstrating the oxidative profile of myofibers. Scale bar, 200 μm is valid for all panels.

Immunohistochemical and SDH activity-based classification of myofiber types is shown in Figure 1 and the morphometric data are summarized in Table 3. Immunoenzyme histochemistry results showed that the prevalent myofiber type in Krškopolje pig LD muscle was type IIb, followed by IIx, IIa, and I myofibers (Figure 1, Table 3). The production system had a notable impact (Cohen's d > 0.8) on the proportion of type I, IIx, and IIb myofibers; however, it was observed to be statistically significant in the case of type I and IIb myofibers only. In comparison to the LD muscle of pigs from conventional indoor husbandry systems (group C), the LD muscle of pigs reared in organic conditions (group O) contained a higher percentage of type I and a lower percentage of type IIb myofibers. This also resulted in a significantly lower glycolytic-to-oxidative myofiber ratio of 1.12 ± 0.24 in the O group compared with 1.45 ± 0.13 in the C group (*p* = 0.03). The husbandry system also notably affected the morphological characteristics of myofibers (Cohen's d > 0.8); however, it was only observed to be significant when the average cross-sectional area of all myofiber types was compared between groups C and O, and the myofiber's cross-sectional areas of LD were found to be smaller in group O than in group C. Similar to fiber ratios, the calculated glycolytic-to-oxidative relative area ratio was also significantly decreased in group O pigs (2.14 ± 0.33 vs. 1.62 ± 0.43 for C and O group, respectively; *p* = 0.014).

**Table 3:** Percentages (%) of myofiber types and their cross-sectional areas (μm^2^) in the longissimus dorsi muscle of Krškopolje pigs according to husbandry.

	**Husbandry**		**Cohen's d**	***P*-value**	**RMSE**
	**C**	**O**			
	**(6)**	**(6)**			
**Myofibre type, %**					
Type I	7.1	8.6^a^	1.3	0.043	1.1
Type IIa	10.9	10.4	0.2	0.751	2.2
Type IIx	22.9	28.5	1.1	0.088	5.2
Type IIb	59.1	52.4^a^	1.7	0.017	4.1
**Myofibre cross-sectional area**, **μm**^**2**^					
Type I	3,851	3,549	0.9	0.140	326
Type IIa	2,842	2,477	0.8	0.204	465
Type IIx	5,875	5,289	1.1	0.087	533
Type IIb	6,866	6,212	1.1	0.096	514
All fibers	5,982	5,326^a^	1.4	0.040	481

Conventional (C) and organic (O) group; number of animals is given in parentheses. Cohen's d denotes effect size; d value above 0.5 is considered medium and above 0.8 a large effect. RMSE, root-mean-square error.

a *-significantly different from the C group (p ≤ 0.05)*.

### Expression of Genes Related to the Contractile and Metabolic Phenotype of Myofibers

The relative expression of genes related to the contractile and metabolic phenotype of myofibers in the LD of Krškopolje pig is shown in Table 4. The system of husbandry strongly and significantly affected the relative mRNA expression levels of MyHC isoforms I, IIa, IIx, and IIb underlying contractile phenotype. The levels of MyHC isoforms I, IIa, and IIx were observed to be significantly (~2-fold) higher, whereas the level of isoform IIb was observed to be significantly (~3.5-fold) lower in the O group than in the C group (Table 4). On the contrary, husbandry did not significantly affect the relative expression of the genes associated with the muscle metabolic phenotypes (Table 4). Despite the lack of statistical significance, it is noticeable that the husbandry system exhibited a medium effect on the relative expression of mRNA in the case of PGC-1α and fatty acid synthase (FASN) (Cohen's d of 0.7 and 0.5, respectively) (Table 4).

**Table 4:** Effect of the production system (husbandry) on the relative mRNA expression of MyHCs and selected genes related to energy metabolism in the longissimus dorsi muscle of Krškopolje pigs.

**Husbandry**						
	**C**	**O**	**Cohen's d**	***p*-value**	**RMSE**	**Fold change**
	**(11)**	**(10)**				**compared to C**
**MyHC**	**ΔCt**	**ΔCt**				
*MyHC I*	−5.3	−4.3^a^	1.4	0.006	0.7	2.0 (2.6–1.5)
*MyHC IIa*	−5.8	−4.4^a^	2.2	<0.001	0.6	2.6 (3.4–2.0)
*MyHC IIx*	−2.5	−1.2	1.8	<0.001	0.7	2.4 (3.1–1.9)
*MyHC IIb*	−2.4	−4.2^a^	1.5	0.005	1.3	−3.5 (−2.4 to −5.1)
**Energy metabolism-related genes**						
*PGC-1*α	−10.9	−11.7	0.7	0.181	1.3	−1.7 (−1.1 to −2.7)
*HK2*	−10.6	−10.2	0.4	0.337	0.8	1.3 (1.6–1.0)
*GYS1*	−8.3	−8.0	0.4	0.400	0.6	1.2 (1.4 to −1.0)
*PPARγ*	−13.4	−13.4	0.0	0.914	0.8	1.0 (1.3 to −1.3)
*LPL*	−7.4	−7.5	0.1	0.764	0.8	−1.1 (1.2 to −1.3)
*FASN*	−10.2	−10.7	0.5	0.239	0.9	−1.4 (−1.0 to −1.9)
*CPT-1B*	−11.5	−11.6	0.2	0.671	0.8	−1.1 (1.2 to −1.5)

Given here are the mean ΔCt values of target transcripts derived using the comparative Ct method (ΔCt = Ct_geometric mean of controls_ – Ct_target transcript_) and the mean fold changes with upper and lower limits in expression for group O pigs in comparison to the group C pigs. A fold-change value <1 was replaced by the negative inverse of the original fold change value.

Conventional (C) and organic (O) group; number of animals is given in parentheses; Cohen's d denotes effect size; d value above 0.5 is considered medium and above 0.8 a large effect. RMSE, root-mean-square error.

a *-significantly different from the C group (p ≤ 0.05)*.

### Correlation Between Histology Based Myofiber Classification and Relative Expression of MyHC Isoforms

**Figure 2:**
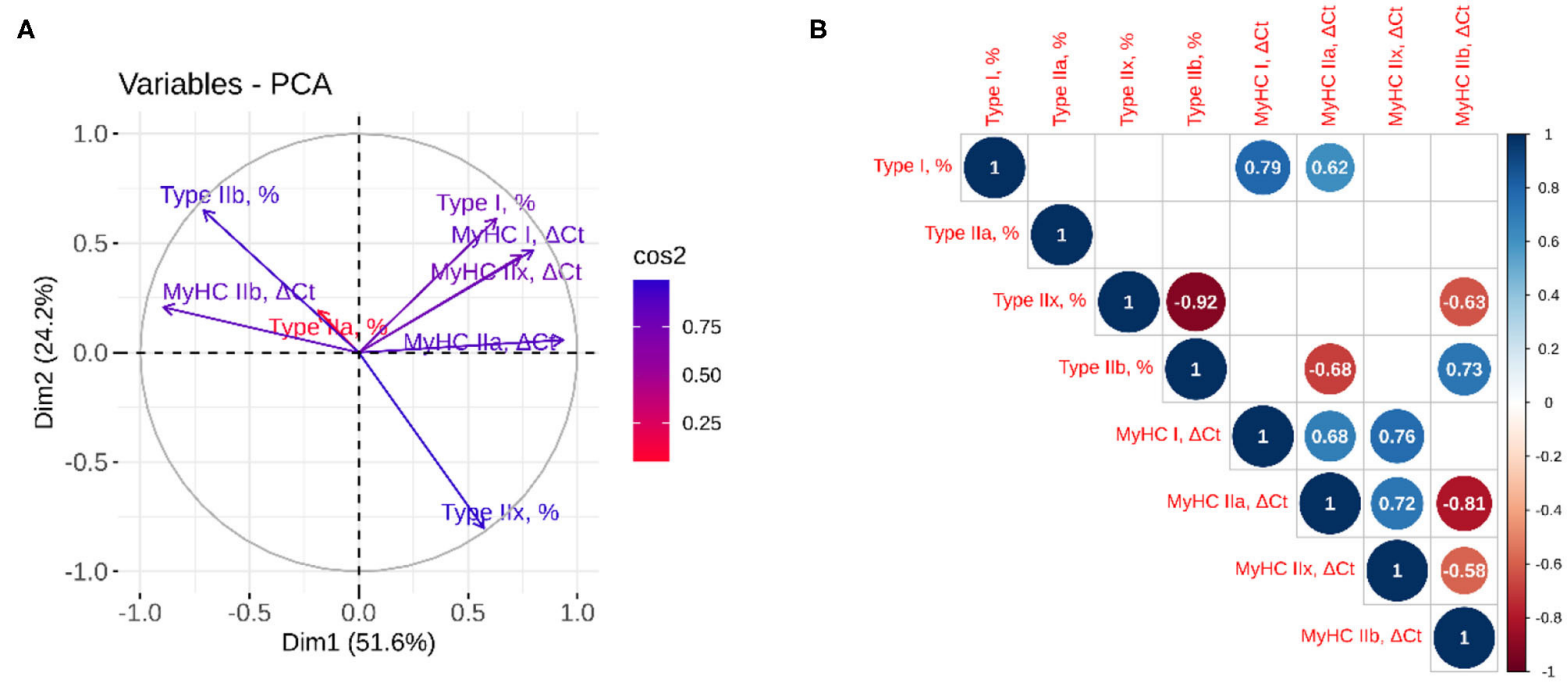
Principal component and correlation analyses between myofiber classification and relative expression of MyHC isoforms. **(A)** The quality of representation was measured using the squared cosine (cos^2^), and the variables that contribute most to the separation of the trait between dimensions 1 and 2 are in blue; **(B)** The correlation analysis between variables. The size and color of the circle denote the strength of the relationship and its direction. Only the significant correlations are represented, and the values of the correlation are given inside the circles.

PCA and correlation analysis were performed to establish the relationship between histology based myofiber classification and the relative expression of MyHC isoforms (Figure 2). The results showed that over 75% of the variation was explained by the first two components, with high quality of representation (cos^2^) for all variables except for the percentage of type IIa myofibers. Together with the correlation analysis, PCA demonstrated that the proportion of type I and IIb myofibers and the mRNA abundance of the corresponding genes (MyHC I and IIb) were well-correlated (*r* = 0.79; *p* = 0.002 and 0.73; *p* = 0.007, respectively). Positive correlation was also observed between the type I myofibers and the *MyHC IIa* expression (*r* = 0.62; *p* = 0.032), whereas the proportion of type IIx myofibers was negatively correlated with the *MyHC IIb* expression (*r* = −0.63; *p* = 0.029). The correlation between the proportion of type IIa and IIx myofibers and the mRNA abundance of the corresponding genes (*MyHC IIa and IIx*) was not significant (*p* > 0.05). The mRNA abundances of MyHCs showed positive correlation of MyHC I with MyHC IIa (*r* = 0.68; *p* = 0.014) and IIx (*r* = 0.72; *p* =0.004), MyHC IIa with IIx (*r* = 0.72; *p* = 0.008) and negative correlation of *MyHC IIb* with the mRNA levels of *MyHC IIa* (*r* = −0.81; *p* = 0.002) and *MyHC IIx* (*r* = −0.58; *p* = 0.047).

### Correlation Between the Relative Expression of Genes Related to Contractile (MyHC Isoforms) and Metabolic Phenotype (Energy Metabolism-Related Genes)

**Figure 3:**
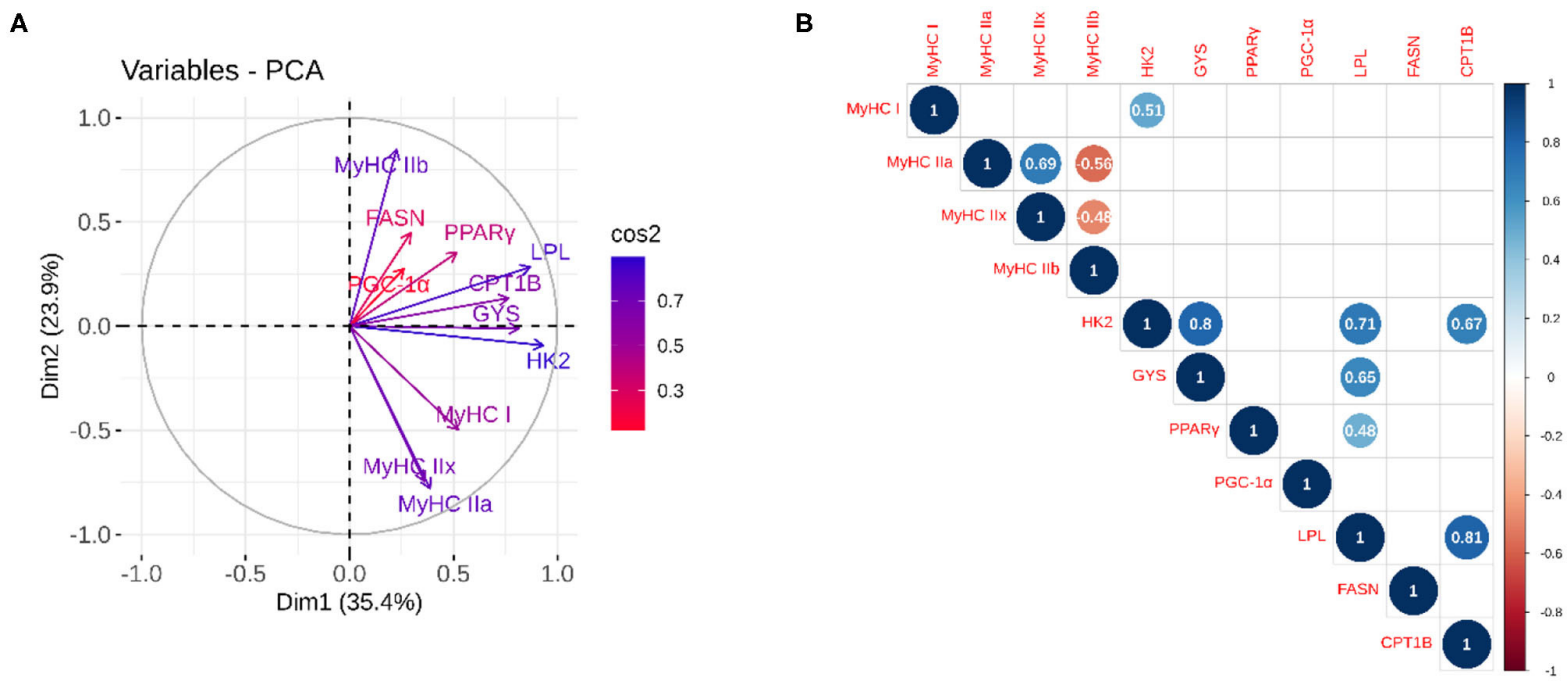
Principal component and correlation analysis between the relative expression of genes related to contractile (*MyHC* isoforms) and metabolic phenotypes (energy metabolism-related genes). **(A)** The quality of representation was measured using the squared cosine (cos^2^), and the variables that contribute most to the separation of the trait between dimensions 1 and 2 are in blue; **(B)** The correlation analysis between variables. The size and color of the circle denote the strength of the relationship and its direction. Only the significant correlations are represented, and the values of the correlation are given inside the circles.

The results of PCA (Figure 3A) showed that 59% of the variation was explained by the first two components, with high association (cos^2^) for all variables except for *FASN, PGC-1*α, and *PPAR*γ. Together with correlation analysis (Figure 3B), it demonstrated good correlation between the mRNA abundances of energy metabolism-related genes, including *hexokinase 2 (*HK2) with *glycogen synthase 1 (GYS)* (*r* = 0.80; *p* = 0.002), *lipoprotein-lipase (LPL)* (*r* = 0.71; *p* ≤ 0.001) and *carnitine palmitoyltransferase 1B (CPT1B)* (*r* = 0.67; *p* ≤ 0.001) and *LPL* with GYS (*r* = 0.65; *p* = 0.002) and *peroxisome proliferator-activated receptor gamma (PPAR*γ*)* (*r* = 0.48; *p* = 0.026) and *CPT1B* (*r* = 0.81; *p* ≤ 0.001). On the contrary, no correlation between muscle contractile and energy metabolism genes was evident, except the correlation between *HK2* and *MyHC* I (*r* = 0.51; *p* = 0.017).

## Discussion

In recent years, autochthonous local pig breeds have attracted increased public interest, as their meat is considered to be more convenient for processing into high quality pork products. Moreover, local breeds are raised under different, but usually more extensive (organic) rearing system regarded to be more environmental- and animal-friendly (13). However, information regarding their skeletal muscle characteristics and related meat quality traits in relation to specific rearing systems is still very limited. Skeletal muscle myofibers are dynamic structures that exhibit high plasticity and undergo a type shift, following an obligatory pathway I ↔ IIa ↔ IIx ↔ IIb (31, 32). Physical exercise, ambient temperature, and nutrition (essential amino acid deprivation) are potential tools that can be combined with genetic factors (breed, genotype) in different rearing systems to affect the morphological and physiological characteristics of myofibers and associated meat quality traits (33). Physical exercise is well-known to influence myofiber characteristics, depending on the type and duration of the exercise. Aerobic conditioning has been shown to increase the proportion of type I fibers in humans (34). In studies on pigs, exercise-induced effects on muscle fiber types are varying or inconsistent (35), reporting either (i) no differences in the proportion of myofiber types (36, 37), (ii) increased percentage of type IIa and IIx myofibers (38), or (iii) increased percentage of type I and IIx (39) myofibers. It is notable that in these studies, a standard myosin ATPase-based method was followed that did not distinguish IIx myofibers. In the present study, we have used an immunoenzyme histochemical approach for myofiber classification in combination with the relative mRNA expression levels of the MyHC isoforms for more precise determination of the contractile pattern. A higher percentage of oxidative type I and lower percentage of type IIb myofibers was observed, resulting in a decrease in glycolytic-to-oxidative myofiber type and relative area ratios, thereby indicating a shift toward an increase in oxidative capacity in the O group of pigs which was clearly substantiated by a strong effect on the relative expression of all MyHC isoforms at the mRNA levels (increased expression of MyHC I, IIa, and IIx, and decreased expression of MyHC IIb). This result is further corroborated by the smaller size of myofibers in O group pigs, as fiber size (but not necessarily fiber type) is related to its oxidative capacity (40). These data are also in good agreement with a recent study that has reported on myofiber type morphological traits and the mRNA expression of MyHCs in the LD muscles of pigs reared either with semi-free grazing or at indoor feeding farms (39). Therefore, in the present study, the reason for a shift to more oxidative metabolism in organic pigs might be increased exercise. However, reports have revealed that the same effect could also be induced by non-exercise means, such as a lower dietary lysine level (41) or dietary butyrate supplementation (42). Therefore, it could be assumed that even in our case, a lower dietary lysine level in the O group (17) could be a contributing factor for the higher percentage of oxidative myofibers and, thus, muscle oxidative capacity. Although, lower dietary lysine levels have been previously correlated with a higher proportion of monounsaturated fatty acids, the LD IMF content did not differ substantially between the groups (17). This argues against the predominant effect of lysine, as it has been reported that the IMF content of the muscle increases with decreasing dietary lysine levels (43).

Earlier studies have provided evidences for the negative influence of fiber hypertrophy on meat quality traits, such as shear force, drip loss, and cooking loss (3, 9). In both groups, the CSA of type IIx and IIb fiber in the LD muscles of Krškopolje pig was observed to be considerably smaller in comparison to modern white pigs, but larger than in wild pigs of comparable age (~7 months) (18). Data derived from immunohistochemistry-based classification of myofiber types and analysis of MyHC mRNA generally showed a strong correlation in the case of MyHC I and IIb, and moderate and weak in the case of MyHC IIa and MyHC IIx, respectively, indicating that there are still difficulties in correctly classifying the latter. In pigs, the SC 71 antibody has been reported to recognize both MyHC isoforms IIa and IIx; however, with different affinities (6). It was also speculated that the reported cross-reactivity of SC 71 with MyHC IIx (6) is due to the abundance of hybrid IIa/IIx myofibers (44). This may explain the problem with the classification of the subset of myofibers, especially those in which two MyHC isoforms, i.e., IIa and IIx or IIx and IIb, are co-expressed at different ratios.

On the basis of an increased oxidative potential of LD in organic pigs, differences were also expected in the expression of some key genes involved in myofiber energy metabolism, as physical activity could be considered one of the most potent physiological stimuli promoting oxidative metabolism in skeletal muscles (45) because of the potential association with meat quality and, in particular, intramuscular fat (IMF) content (46–49). Therefore, the expression of selected energy metabolism-related genes involved in carbohydrate and lipid metabolism pathways was addressed in the present study. In the skeletal muscle glycolytic cycle, the phosphorylation of glucose to glucose-6-phosphate is controlled by hexokinase 2 (HK2), which is a key enzyme of glycolysis in pig skeletal muscle (50). Another promising candidate gene for traits related to skeletal muscle metabolism in pigs is the *GYS1*, which encodes glycogen synthase (51). The mRNA levels of these two enzymes (*HK2, GYS1*) were shown to be significantly higher in fast glycolytic-type muscles than in slow oxidative-type pig muscles (48). Although in the present study, the husbandry system demonstrated no statistically significant effect on either *HK2* or *GYS1* expression, a positive correlation between the *HK2* and *MyHC I* expression is quite intriguing as, to the best of our knowledge, the upregulation of enzymes involved in carbohydrate metabolism has only been reported in slow-twitch type I aging human myofibers (52).

With respect to lipid metabolism-related genes, the increased oxidative potential of LD in organic pigs was not accompanied by an increase in the relative expression of the studied lipid metabolism genes, including *PGC-1*α, *PPAR*γ, *LPL, CPT1B*, and *FASN. PGC-1*α has a clear impact on metabolism through its effects on many downstream target genes important for oxidative metabolism (23). *PGC-1*α has been identified as a cold- and exercise-induced coactivator important for mitochondrial biogenesis/function (53, 54), myofiber transformation from the fast- to the slow-twitch type (55), and myoblast differentiation/maturation (56). A recent study has also suggested *PGC-1*α upregulation to be a possible molecular mechanism underlying the butyrate diet-promoted increase in the slow-twitch myofiber type in finishing pigs (42). Considering the aforementioned results, an increased expression level of *PGC-1*α was anticipated in organic pigs; however, its level tended to be lower than that in conventionally reared pigs. The *PGC-1*α expression data could be interpreted with the aid of some other reports, revealing (i) a lack of association between the *PGC-1*α level and the muscle oxidative capacity (SDH activity) in human myofibers (57); (ii) the predominant post-translational regulation of PGC-1α activity (45, 58); (iii) a lack of dietary lysine level effects on *PGC-1*α expression in the LD muscle, but not in oxidative-type rhomboid muscle (41); and (iv) age-related variations in *PGC-1*α expression between wild and conventionally reared domestic pigs, as the *PGC-1*α level increased only during the early postnatal period, and not in adult wild pigs (22).

Peroxisome proliferator-activated receptor gamma (*PPAR*γ or *PPARG*) is mainly present in adipose tissue, where it regulates fatty acid storage and glucose metabolism, such as lipid uptake and adipogenesis by fat cells, by triggering genes, including *LPL*, which stimulates fatty acid uptake in muscle tissues (59). Correlation between *PPAR*γ expression and intramuscular fat deposition has also been previously reported in the Chinese indigenous fatty pig (Laiwu pig), known for its high meat quality (60). We observed a positive co-expression of these two genes; however, no effect of the husbandry system was observed. We also failed to find any differences in the relative expression levels of the *CPT1B* that facilitates lipid utilization through regulation of mitochondrial fatty acid oxidation (61), though we found a positive correlation between *PPAR*γ and *CPT1B*. The lipid content of slow type I myofibers was observed to be three times to that of glycolytic IIb myofibers (62), and a positive correlation between the expression of *MyHC I, IIa*, and *IIx*, and a negative correlation between the expression of *MyHC IIb* and intramuscular fat content, have been previously reported (63). Thus, it was assumed that the relative expression levels of individual *MyHCs* and the expression levels of lipid metabolism-related genes could be correlated that is not confirmed by the results of the present study. Although a higher percentage of oxidative (SDH-positive) myofibers that utilize lipids as the major energy source, was established for organic pigs, there were no differences in the expression levels of key lipid metabolism-related genes. The effect of organic rearing on intramuscular fat content was moderate (Cohen's d being 0.5), but insignificant (17), that can be related to the predominant localization of lipids in the form of adipose tissue between myofibers (39, 64). Interestingly, the size of myofibers was strongly negatively correlated with intramuscular fat content, supporting the hypothesis that fiber size is negatively related to oxidative capacity (40). A weak correlation between the expression of *MyHC* genes and energy metabolism-related genes corroborates the hypothesis that in pig muscle, contractile protein content is transcriptionally directly regulated by the *MyHC* genes and can be uncoupled from energy metabolism genes (6, 65). The lack of effect of rearing on the expression of energy metabolism-related genes also corroborates with the results from previously reported chemical analyses showing no differences in the LD muscle IMF content between O and C group (17). Therefore, it could also be assumed that due to the predominance of the glycolytic fibers (> 50% in both groups), a small, i.e., ~1% increase in type I fibers in the O group of pigs, is not sufficient to exert a significant effect on the relative expression of the studied lipid metabolism-related genes.

To summarize, our results showed that an organic production system provides pigs with outdoor space, thus encouraging spontaneous physical activity, resulting in a shift toward the oxidative (SDH-positive) myofiber types, downregulation of *MyHC IIb*, and an upregulation of *MyHC I, IIa*, and *IIx* in the LD of Krškopolje pigs. However, non-exercise/dietary effects, such as lower dietary lysine, in organically grown pigs cannot be excluded. In contrast, the effect of rearing on the expression of energy metabolism-related genes and their correlation with *MyHCs* expression was less evident. Therefore, it can be concluded that the conventional system is less than ideal for the Krškopolje pig breed as it would result in an increased hypertrophic potential of myofibers and higher proportion of type IIb myofibers, known to be negatively correlated with some meat quality traits such as water-holding capacity, pH, and tenderness.

## Data Availability Statement

All datasets presented in this study are included in the article/supplementary material.

## Ethics Statement

Animal procedures were carried out in accordance with the Slovenian Animal Protection Act (Official Gazette of the Republic of Slovenia, No. 43/2007) and were not subject to ethical protocols according to Directive 2010/63/EU on the protection of animals used for scientific purposes. The study was undertaken with full owner compliance within normal running on the farm and muscle tissue samples were collected after the slaughter in a commercial abattoir. The Veterinary faculty is an approved establishment by the Veterinary Administration of the Republic of Slovenia (Approval No. SI B 07-22-25) for use of animal by-products C2 (Category 2 1069/2009/ES) and C3 (Category 3 1069/2009/ES) for research purpose.

## Author Contributions

GF and MČ-P contributed conception and design of the study. MV, MŠ, NB-L, UT, JB, and GF performed sample collection and analysis. MČ-P and KP performed the statistical analysis. GF wrote the first draft of the manuscript. MV and MČ-P wrote sections of the manuscript. All authors contributed to manuscript revision, read and approved the submitted version.

## Conflict of Interest

The authors declare that the research was conducted in the absence of any commercial or financial relationships that could be construed as a potential conflict of interest.

## References

[1] Joo ST Kim GD Hwang YH Ryu YC. Control of fresh meat quality through manipulation of muscle fiber characteristics. Meat Sci. (2013) 95:828–36. 10.1016/j.meatsci.2013.04.044 23702339

[2] Lee SH Joo ST Ryu YC. Skeletal muscle fiber type and myofibrillar proteins in relation to meat quality. Meat Sci. (2010) 86:166–70. 10.1016/j.meatsci.2010.04.040 20605337

[3] Listrat A Lebret B Louveau I Astruc T Bonnet M Lefaucheur L. How muscle structure and composition influence meat and flesh quality. ScientificWorldJournal. (2016) 2016:3182746. 10.1155/2016/3182746 27022618 PMC4789028

[4] Lefaucheur L Milan D Ecolan P Le Callennec C. Myosin heavy chain composition of different skeletal muscles in large white and meishan pigs. J Anim Sci. (2004) 82:1931–41. 10.2527/2004.8271931x 15309939

[5] Pellegrino MA Canepari M Rossi R D'Antona G Reggiani C Bottinelli R. Orthologous myosin isoforms and scaling of shortening velocity with body size in mouse, rat, rabbit and human muscles. J Physiol. (2003) 546:677–89. 10.1113/jphysiol.2002.027375 12562996 PMC2342590

[6] Lefaucheur L Ecolan P Plantard L Gueguen N. New insights into muscle fiber types in the pig. J Histochem Cytochem. (2002) 50:719–30. 10.1177/002215540205000513 11967283

[7] Gentry JG McGlone JJ Miller MF Blanton JR Jr. Diverse birth and rearing environment effects on pig growth and meat quality. J Anim Sci. (2002) 80:1707–15. 10.2527/2002.8071707x 12162637

[8] Jing L Hou Y Wu H Miao Y Li X Cao J. Transcriptome analysis of mRNA and miRNA in skeletal muscle indicates an important network for differential residual feed intake in pigs. Sci Rep. (2015) 5:11953. 10.1038/srep11953 26150313 PMC4493709

[9] Lefaucheur L Lebret B Ecolan P Louveau I Damon M Prunier A. Muscle characteristics and meat quality traits are affected by divergent selection on residual feed intake in pigs. J Anim Sci. (2011) 89:996–1010. 10.2527/jas.2010-3493 21148787

[10] Olsson V Andersson K Hansson I Lundstrom K. Differences in meat quality between organically and conventionally produced pigs. Meat Sci. (2003) 64:287–97. 10.1016/S0309-1740(02)00200-0 22063015

[11] Tribout T Caritez JC Gruand J Bouffaud M Guillouet P Billon Y. Estimation of genetic trends in French large white pigs from 1977 to 1998 for growth and carcass traits using frozen semen. J Anim Sci. (2010) 88:2856–67. 10.2527/jas.2009-2356 20495129

[12] Ruusunen M Puolanne E. Histochemical properties of fibre types in muscles of wild and domestic pigs and the effect of growth rate on muscle fibre properties. Meat Sci. (2004) 67:533–9. 10.1016/j.meatsci.2003.12.008 22061529

[13] Candek-Potokar M Batorek-Lukač N TomaŽin U Škrlep M Nieto R Analytical review of productive performance of local pig breeds. In: Candek-Potokar M, Linan RMN, editors. European Local Pig Breeds–Diversity and Performance A Study of Project Treasure. London: IntechOpen (2019). p. 281–303. 10.5772/intechopen.84214

[14] Bonneau M Lebret B. Production systems and influence on eating quality of pork. Meat Sci. (2010) 84:293–300. 10.1016/j.meatsci.2009.03.013 20374788

[15] Batorek-Lukač N TomaŽin U Škrlep M Kastelic A Poklukar K Candek-Potokar M Krškopoljski prašič (Krškopolje pig). In: Candek-Potokar M, Linan RMN, editors. European Local Pig Breeds - Diversity and Performance A Study of Project TREASURE. London: IntechOpen (2019). p. 1–15. 10.5772/intechopen.83767

[16] Candek-Potokar M Žlender B Kramar Z Šegula B Fazarinc G Uršič M Evaluation of slovene local pig breed krškopolje for carcass and meat quality. Czech J Anim Sci. (2003) 8:120–8.

[17] TomaŽin U Batorek-Lukač N Škrlep M Prevolnik-Povše M Candek-Potokar M. Meat and fat quality of krskopolje pigs reared in conventional and organic production systems. Animal. (2019) 13:1103–10. 10.1017/S1751731118002409 30289382

[18] Fazarinc G Vrecl M Škorjanc D Cehovin T Candek-Potokar M. Dynamics of myosin heavy chain isoform transition in the longissimus muscle of domestic and wild pigs during growth: a comparative study. Animal. (2017) 11:164–74. 10.1017/S1751731116001312 27345286

[19] Schiaffino S Gorza L Sartore S Saggin L Ausoni S Vianello M. Three myosin heavy chain isoforms in type 2 skeletal muscle fibres. J Muscle Res Cell Motil. (1989) 10:197–205. 10.1007/BF01739810 2547831

[20] Graziotti GH Menendez JMR Rios CM Cossu ME Bosco A Affricano NO Relationship between myosin isoforms and meat quality traits in pig semitendinosus neuromuscular compartments. Asian Aust J Anim Sci. (2011) 24:125–9. 10.5713/ajas.2011.10237

[21] Nachlas MM Tsou KC de Souza E Cheng CS Seligman AM. Cytochemical demonstration of succinic dehydrogenase by the use of a new p-nitrophenyl substituted ditetrazole. J Histochem Cytochem. (1957) 5:420–36. 10.1177/5.4.420 13463314

[22] Vrecl M Cotman M Ursic M Candek-Potokar M Fazarinc G. Age-dependent expression of MyHC isoforms and lipid metabolism-related genes in the longissimus dorsi muscle of wild and domestic pigs. Animals. (2019) 9:10. 10.3390/ani9010010 30597908 PMC6357074

[23] Erkens T van Poucke M van Desompele J Goossens K van Zeveren A Peelman LJ. Development of a new set of reference genes for normalization of real-time RT-PCR data of porcine backfat and longissimus dorsi muscle, and evaluation with PPARGC1A. BMC Biotechnol. (2006) 6:41. 10.1186/1472-6750-6-41 17026777 PMC1609116

[24] Wimmers K Ngu NT Jennen DG Tesfaye D Murani E Schellander K. Relationship between myosin heavy chain isoform expression and muscling in several diverse pig breeds. J Anim Sci. (2008) 86:795–803. 10.2527/jas.2006-521 18156349

[25] Sullivan GM Feinn R. Using effect size-or why the P value is not enough. J Grad Med Educ. (2012) 4:279–82. 10.4300/JGME-D-12-00156.1 23997866 PMC3444174

[26] R Core Team R: A Language and Environment for Statistical Computing. Vienna: R Foundation for Statistical Computing (2013).

[27] Lê S Josse J Husson F FactoMineR: an R package for multivariate analysis. J Stat Softw. (2008) 25:1–18. 10.18637/jss.v025.i01

[28] Kassambara A Mundt F Factoextra: Extract and Visualize the Results of Multivariate Data Analyses. Vienna: R Package Version 1.0.5 (2017).

[29] Harrell FE With Contributions from Charles Dupont and Many Others. Hmisc: Harrell Miscellaneous, R Package Version 4.2-0 (2019).

[30] Wei T Simko V Levy M Xie Y Jin Y Zemla J R Package Corrplot: Visualization of a Correlation Matrix (Version 0.84). Vienna (2017).

[31] Pette D Staron RS. Myosin isoforms, muscle fiber types, and transitions. Microsc Res Tech. (2000) 50:500–9. 10.1002/1097-0029(20000915)50:6<500::AID-JEMT7>3.0.CO;2-7 10998639

[32] Schiaffino S Reggiani C. Myosin isoforms in mammalian skeletal muscle. J Appl Physiol. (1994) 77:493–501. 10.1152/jappl.1994.77.2.493 8002492

[33] Lefaucheur L. A second look into fibre typing–relation to meat quality. Meat Sci. (2010) 84:257–70. 10.1016/j.meatsci.2009.05.004 20374784

[34] Allemeier CA Fry AC Johnson P Hikida RS Hagerman FC Staron RS. Effects of sprint cycle training on human skeletal muscle. J Appl Physiol. (1994) 77:2385–90. 10.1152/jappl.1994.77.5.2385 7868459

[35] Gondret F Combes S Lefaucheur L Lebret B. Effects of exercise during growth and alternative rearing systems on muscle fibers and collagen properties. Reprod Nutr Dev. (2005) 45:69–86. 10.1051/rnd:2005003 15865057

[36] Essen-Gustavsson B Lundstrom K Larsson G Lindholm A Nordin AC Hansson I The effect during growth of moderate exercise on muscle metabolic characteristics *in vivo* and relation to meat quality and sensory properties. In: Chandler CS, Thornton RF, editors. 34th International Congress of Meat Science & Technology. Brisbane, QLD: IcoMST (1988). p. 27–30.

[37] Gentry JG McGlone JJ Blanton JR Jr Miller MF. Impact of spontaneous exercise on performance, meat quality, and muscle fiber characteristics of growing/finishing pigs. J Anim Sci. (2002) 80:2833–9. 10.2527/2002.80112833x 12462250

[38] Petersen JS Henckel P Oksbjerg N Sørensen MT Adaptations in muscle fibre characteristics induced by physical activity in pigs. Anim Sci. (1998) 66:733–40. 10.1017/S1357729800009310

[39] Qi K Men X Wu J Xu Z. Rearing pattern alters porcine myofiber type, fat deposition, associated microbial communities and functional capacity. BMC Microbiol. (2019) 19:181. 10.1186/s12866-019-1556-x 31387544 PMC6683424

[40] van Wessel T de Haan A van der Laarse WJ Jaspers RT The muscle fiber type-fiber size paradox: hypertrophy or oxidative metabolism? Eur J Appl Physiol. (2010) 110:665–94. 10.1007/s00421-010-1545-0 20602111 PMC2957584

[41] Katsumata M Matsumoto M Kobayashi S-I Kaji Y Reduced dietary lysine enhances proportion of oxidative fibers in porcine skeletal muscle. Anim Sci J. (2008) 79:347–53. 10.1111/j.1740-0929.2008.00536.x

[42] Zhang Y Yu B Yu J Zheng P Huang Z Luo Y. Butyrate promotes slow-twitch myofiber formation and mitochondrial biogenesis in finishing pigs via inducing specific microRNAs and PGC-1alpha expression1. J Anim Sci. (2019) 97:3180–92. 10.1093/jas/skz187 31228349 PMC6667260

[43] Wang T Crenshaw MA Regmi N Rude BJ Hasan S Sukumaran AT Effects of dietary lysine level on the content and fatty acid composition of intramuscular fat in late-stage finishing pigs. Can J Anim Sci. (2017) 98:241–9. 10.1139/cjas-2017-0083

[44] Toniolo L Patruno M Maccatrozzo L Pellegrino MA Canepari M Rossi R. Fast fibres in a large animal: fibre types, contractile properties and myosin expression in pig skeletal muscles. J Exp Biol. (2004) 207:1875–86. 10.1242/jeb.00950 15107442

[45] Popov DV. Adaptation of skeletal muscles to contractile activity of varying duration and intensity: the role of PGC-1alpha. Biochemistry. (2018) 83:613–28. 10.1134/S0006297918060019 30195320

[46] Erkens T van Desompele J van Zeveren A Peelman LJ. Correlation between porcine PPARGC1A mRNA expression and its downstream target genes in backfat and longissimus dorsi muscle. J Appl Genet. (2009) 50:361–9. 10.1007/BF03195694 19875886

[47] Huang YN Ao QW Jiang QY Guo YF Lan GQ Jiang HS. Comparisons of different myosin heavy chain types, AMPK, and PGC-1alpha gene expression in the longissimus dorsi muscles in bama xiang and landrace pigs. Genet Mol Res. (2016) 15:gmr8379. 10.4238/gmr.15028379 27421023

[48] Shen LY Luo J Lei HG Jiang YZ Bai L Li MZ. Effects of muscle fiber type on glycolytic potential and meat quality traits in different tibetan pig muscles and their association with glycolysis-related gene expression. Genet Mol Res. (2015) 14:14366–78. 10.4238/2015.November.13.22 26600496

[49] Zhang C Luo JQ Zheng P Yu B Huang ZQ Mao XB. Differential expression of lipid metabolism-related genes and myosin heavy chain isoform genes in pig muscle tissue leading to different meat quality. Animal. (2015) 9:1073–80. 10.1017/S1751731115000324 25716066

[50] Wang J Deng C-Y Xiong Y-Z Zuo B Cheng H-C Li F Sequencing, polymorphism and expression profile analysis of porcine hexokinase II (HK2) gene. Agric Sci China. (2006) 5:384–9. 10.1016/S1671-2927(06)60065-5

[51] Zuo B Xiong YZ Deng CY Su YH Wang J Lei MG. Polymorphism, linkage mapping and expression pattern of the porcine skeletal muscle glycogen synthase (GYS1) gene. Anim Genet. (2005) 36:254–7. 10.1111/j.1365-2052.2005.01286.x 15932409

[52] Murgia M Toniolo L Nagaraj N Ciciliot S Vindigni V Schiaffino S. Single muscle fiber proteomics reveals fiber-type-specific features of human muscle aging. Cell Rep. (2017) 19:2396–409. 10.1016/j.celrep.2017.05.054 28614723

[53] Safdar A Little JP Stokl AJ Hettinga BP Akhtar M Tarnopolsky MA. Exercise increases mitochondrial PGC-1α content and promotes nuclear-mitochondrial cross-talk to coordinate mitochondrial biogenesis. J Biol Chem. (2011) 286:10605–17. 10.1074/jbc.M110.211466 21245132 PMC3060512

[54] Wu Z Puigserver P Andersson U Zhang C Adelmant G Mootha V. Mechanisms controlling mitochondrial biogenesis and respiration through the thermogenic coactivator PGC-1. Cell. (1999) 98:115–24. 10.1016/S0092-8674(00)80611-X 10412986

[55] Lin J Wu H Tarr PT Zhang CY Wu Z Boss O. Transcriptional co-activator PGC-1α drives the formation of slow-twitch muscle fibres. Nature. (2002) 418:797–801. 10.1038/nature00904 12181572

[56] Lin Y Zhao Y Li R Gong J Zheng Y Wang Y PGC-1α is associated with C2C12 myoblast differentiation. Cent Eur J Biol. (2014) 9:1030–6. 10.2478/s11535-014-0341-y

[57] Gouspillou G Sgarioto N Norris B Barbat-Artigas S Aubertin-Leheudre M Morais JA. The relationship between muscle fiber type-specific PGC-1aα content and mitochondrial content varies between rodent models and humans. PLoS ONE. (2014) 9:e103044. 10.1371/journal.pone.0103044 25121500 PMC4133187

[58] Julien IB Sephton CF Dutchak PA. Metabolic networks influencing skeletal muscle fiber composition. Front Cell Dev Biol. (2018) 6:125. 10.3389/fcell.2018.00125 30324104 PMC6172607

[59] Tontonoz P Spiegelman BM. Fat and beyond: the diverse biology of PPARgamma. Annu Rev Biochem. (2008) 77:289–312. 10.1146/annurev.biochem.77.061307.091829 18518822

[60] Cui J Chen W Liu J Xu T Zeng Y. Study on quantitative expression of PPARgamma and ADRP in muscle and its association with intramuscular fat deposition of pig. Springerplus. (2016) 5:1501. 10.1186/s40064-016-3187-0 27652074 PMC5014771

[61] Lee WJ Kim M Park HS Kim HS Jeon MJ Oh KS. AMPK activation increases fatty acid oxidation in skeletal muscle by activating PPARalpha and PGC-1. Biochem Biophys Res Commun. (2006) 340:291–5. 10.1016/j.bbrc.2005.12.011 16364253

[62] de Feyter HM Schaart G Hesselink MK Schrauwen P Nicolay K Prompers JJ. Regional variations in intramyocellular lipid concentration correlate with muscle fiber type distribution in rat tibialis anterior muscle. Magn Reson Med. (2006) 56:19–25. 10.1002/mrm.20924 16767761

[63] Hu H Wang J Zhu R Guo J Wu Y. Effect of myosin heavy chain composition of muscles on meat quality in Laiwu pigs and Duroc. Sci China C Life Sci. (2008) 51:127–32. 10.1007/s11427-008-0016-x 18239890

[64] Picard B Berri C Lefaucheur L Molette C Sayd T Terlouw C. Skeletal muscle proteomics in livestock production. Brief Funct Genomics. (2010) 9:259–78. 10.1093/bfgp/elq005 20308039

[65] Park SK Gunawan AM Scheffler TL Grant AL Gerrard DE. Myosin heavy chain isoform content and energy metabolism can be uncoupled in pig skeletal muscle. J Anim Sci. (2009) 87:522–31. 10.2527/jas.2008-1269 18820156

